# Pex8, a Fungal Specific Peroxin, Regulates Peroxisome Biogenesis and Pathogenicity in the Cucumber Anthracnose Fungus *Colletotrichum orbiculare*

**DOI:** 10.3390/jof12040248

**Published:** 2026-03-30

**Authors:** Xinhe Wang, Jing Wang, Shendan Yu, Yingying Cai, Yanxi Lin, Zhen Zhang, Muhammad Noman, Haiping Qiu, Zhongna Hao, Rongyao Chai, Yanli Wang, Lin Li, Ling Li, Jiaoyu Wang

**Affiliations:** 1 Zhejiang Key Laboratory of Biology and Ecological Regulation of Crop Pathogens and Insects, College of Advanced Agricultural Sciences, Zhejiang A&F University, Hangzhou 311300, China; 2 State Key Laboratory for Quality and Safety of Agro-Products, Key Laboratory of Agricultural Microbiome (MARA), Key Laboratory of Agricultural Microbiome of Zhejiang Province, Key Laboratory of Biotechnology in Plant Protection of MARA, Institute of Plant Protection and Microbiology, Zhejiang Academy of Agricultural Sciences, Hangzhou 310021, China; 3 College of Biotechnology and Bioengineering, Zhejiang University of Technology, Hangzhou 310014, China

**Keywords:** *Colletotrichum orbiculare*, peroxisome, appressorium, *CoPEX8*, pathogenicity

## Abstract

Peroxisomes are ubiquitous eukaryotic organelles that play critical roles in the infection processes of many plant pathogenic fungi. Peroxisome biogenesis depends on peroxins encoded by *PEX* genes. Pex8 is a fungus-specific peroxin present only in yeasts and filamentous fungi. In this study, we investigated the function of *CoPEX8* in the cucumber anthracnose fungus *Colletotrichum orbiculare* using targeted gene deletion. Fluorescence microscopy using red fluorescent protein fused to peroxisomal targeting signal 1 (PTS1) showed that matrix protein import was abolished in the Δ*Copex8* mutant. Compared with the wild-type strain, the Δ*Copex8* mutant lacked detectable peroxisomes and exhibited severe defects in melanin production, fatty acid utilization, cell wall integrity, osmotic stress tolerance, and reactive oxygen species (ROS) scavenging. Deletion of *CoPEX8* also reduced conidiation and impaired appressorium formation. Pathogenicity assays on cucumber leaves revealed that lesions produced by the Δ*Copex8* mutant were significantly smaller than those caused by the wild-type strain. These results demonstrate that *CoPEX8* is indispensable for peroxisome biogenesis and is essential for both development and virulence of *C. orbiculare*.

## 1. Introduction

*Colletotrichum orbiculare* is a hemibiotrophic fungal pathogen that causes anthracnose on cucurbits, resulting in substantial economic losses worldwide. The pathogen infects all aerial plant parts, producing characteristic sunken lesions that reduce both yield and marketable quality [1]. In major watermelon-producing regions of the United States and India, yield losses commonly range from 30 to 80% under favorable disease conditions, with post-harvest losses further exacerbated by shortened storage life [2,3,4]. The cucumber industry experiences a 15–20% yield reduction due to this disease [5]. Successful infection by *C. orbiculare* relies on a sophisticated developmental program involving conidial germination, appressorium formation, melanization, mechanical penetration, and host colonization. Upon spore germination, *C. orbiculare* differentiates its germ tube tip into a specialized appressorium. This structure adheres to the host surface through secretion from its contact site and forms a conical penetration peg on the distal side to invade the host.

Appressorium melanization is critical for pathogenicity [6]. During its differentiation, melanin biosynthesis is activated, and the resulting oxidized polymers are deposited in the cell wall to form a sealed osmotic chamber, enabling turgor pressure buildup. Melanized appressoria generate enormous turgor pressure, enabling direct penetration of the host cuticle. Following cuticle penetration, the peg differentiates into primary infection hyphae [7]. These hyphae secrete the cell wall-degrading enzymes pectate lyase (PL1) and cellulase (CBH6) to facilitate invasion. Subsequently, the melanin layer is degraded via autophagy, liberating stored carbon sources to fuel further hyphal expansion and ultimately leading to disease progression in the host [8]. This process requires mobilization of stored carbon reserves (glycogen and lipids) and is supported by peroxisome-mediated β-oxidation of fatty acids, the glyoxylate cycle, and ROS detoxification [9].

Peroxisomes are single-membrane-bound organelles present in nearly all eukaryotes. Their biogenesis and function depend on more than 30 peroxins encoded by *PEX* genes. Peroxisomes play a critical role in fungal development and virulence [10]. Several key metabolic processes occur within peroxisomes, including fatty acid β-oxidation, catalase-mediated decomposition of hydrogen peroxide, and glyoxylate metabolism. In plant pathogenic fungi such as *Magnaporthe oryzae* and *Colletotrichum* spp., peroxisomal metabolism is essential for appressorium maturation and pathogenicity [11,12,13]. Moreover, peroxisomes contain enzymes directly involved in the biosynthesis of toxins and melanin. Mutations in genes related to fatty acid metabolism—such as *MoPCS60* and *MoFAS1* in *M. oryzae*—disrupt lipid metabolism and impair melanin production, further highlighting the importance of peroxisomes in the pathogenicity of plant pathogenic fungi [14,15].

Peroxisome biogenesis and function depend on a highly conserved, multi-component import machinery for matrix proteins [16]. In this system, proteins bearing a C-terminal PTS1 or N-terminal PTS2 signal are recognized in the cytosol by the receptors Pex5 and Pex7, respectively [17]. The receptor–cargo complexes dock onto the peroxisomal membrane through the Pex13–Pex14 complex, with Pex17 (termed Pex33 in most filamentous fungi) providing essential support. After cargo translocation and release, receptor recycling is achieved via monoubiquitination of Pex5, catalyzed by the E2 ubiquitin-conjugating enzyme Pex4 (anchored by Pex22) in conjunction with the RING-finger E3 ligase complex (Pex2–Pex10–Pex12) [10,18,19]. The monoubiquitinated Pex5 is subsequently extracted from the membrane in an ATP-dependent manner by the AAA+ ATPase complex Pex1Pex6 (anchored by Pex15 or Pex26 in fungi) [20]. Following deubiquitination in the cytosol, Pex5 is released for additional rounds of import [19].

In yeasts, Pex8 is required for matrix protein import and receptor recycling [21]. As a peroxisomal membrane protein, it plays a crucial role in cargo release and complex formation on the peroxisomal membrane. Studies have shown that Pex8 is exclusively localized within peroxisomes and is involved in the active release of cargo proteins, particularly in collaboration with the receptor Pex5, which is responsible for recognizing peroxisomal targeting signals [22]. In *Yarrowia lipolytica*, Pex8 forms a specific complex with the cytosolic receptor Pex20 and directly facilitates the membrane docking and subsequent import of thiolase [23]. In *Saccharomyces cerevisiae*, deletion of the *PEX8* gene leads to the cytosolic accumulation of both PTS1-type and PTS2-type matrix proteins, as well as the formation of “ghost” peroxisomes–membrane structures that lack matrix content. This underscores Pex8’s critical role in the matrix protein import process [24]. Our previous study revealed that *BcPEX8* is indispensable for the development and pathogenicity of *Botrytis cinerea* [25]. However, its role in other filamentous plant pathogens has remained largely unexplored.

Although the function of *PEX8* has been studied in yeast, its biological role in peroxisome biogenesis, fungal development, and virulence of *C. orbiculare* has not been determined. In this study, we identified and characterized the roles of *CoPEX8* by knocking out this gene in *C. orbiculare.* CoPex8 is localized in the peroxisome membrane and required for the PTS1-dependent matrix protein import and peroxisome biogenesis. We revealed that *CoPEX8* participates in lipid metabolism, stress tolerance, appressorium function, and full virulence of *C. orbiculare*.

## 2. Materials and Methods

### 2.1. Fungal Strains and Culture Conditions

The wild-type strain of *C. orbiculare* (104-T) and all derived mutants were maintained on complete medium (CM) [26] at 25 °C for 9 days [12]. Transformants were generated by *Agrobacterium tumefaciens*-mediated transformation (*At*MT) [27].

### 2.2. Sequence and Phylogenetic Analysis

The *CoPEX8* open reading frame was identified by BLASTp 2.14.1 using Bcpex8 (BCIN_16g01260) from *Botrytis cinerea* as a query. Multiple sequence alignment was performed with GeneDoc 2.7, and a neighbor-joining phylogenetic tree was constructed using MEGA version 10.0.5 software.

### 2.3. Generation of ΔCopex8 Mutants and Complemented Strains

Upstream and downstream flanking regions (~1.5 kb each) of *CoPEX8* were amplified and assembled with a hygromycin resistance cassette into the binary vector pBIG4MR. The resulting knockout construct was introduced into the wild-type strain via *At*MT. Putative Δ*Copex8* mutants were confirmed by PCR and gel electrophoresis.

To complement the mutation, the full-length sequence of the *CoPEX8* gene was amplified using the primer pair CoPEX8-F/CoPEX8-R. The amplified fragment was inserted into the XbaI/SalI restriction sites of the vector pKD5-GFP to construct the complementation vector, which was subsequently introduced into the Δ*Copex8* mutant. Sulfonylurea-resistant transformants were screened, and PCR verification was performed to identify successfully complemented strains. The confirmed complementation strains were used for subsequent phenotypic analysis. The PCR primers used in this study are listed in Table S1.

### 2.4. Fluorescence Microscopy Observation

Peroxisomes were visualized using mCherry-PTS1 or GFP-PTS1 [28]. For CoPex8 localization, the coding sequence was fused to GFP under control of the *M. oryzae* MPG1 promoter. Fluorescence was examined with an Olympus FV3000 confocal microscope (Olympus Corporation, Tokyo, Japan) (excitation 488 nm for GFP, 587 nm for mCherry).

### 2.5. Transmission Electron Microscope (TEM) Observations

Fresh mycelium (~50–100 mg) was fixed in 2.5% (*v*/*v*) glutaraldehyde in 0.1 M phosphate buffer (pH 7.2) and left overnight at 4 °C. Samples were then thoroughly washed three times with 0.1 M phosphate buffer (PBS, pH 7.2), each wash lasting 10–15 min. Samples were then post-fixed in 1% (*w*/*v*) osmium tetroxide in the same buffer at room temperature for 1–2 h, followed by three additional PBS washes (15 min each). Subsequently, samples were dehydrated through a graded ethanol series (50–90%; 15 min per step) and pure acetone (15 min each). The samples were fixed in Spurr resin overnight at room temperature. Subsequently, ultrathin sections (70–100 nm thick) were cut using a Leica UC6 ultramicrotome (Leica Microsystems GmbH, Wetzlar, Germany)., stained with uranyl acetate, and observed using a TEM (H-7650, Hitachi, Tokyo, Japan).

### 2.6. Pathogenicity Assay

*C. orbiculare* wild-type and mutant strains were cultured on CM for 7–8 days at 28 °C. Conidia were harvested by gentle scraping with a sterile spreader and suspended in sterile distilled water to a final concentration of 1 × 10^6^ conidia/mL. Healthy detached cucumber leaves were inoculated with conidial suspension (20 μL). Leaf surfaces inoculated with sterile distilled water were used as controls. Inoculated leaves were maintained in a growth chamber at 25 °C under a 12 h light and 12 h dark photoperiod. Disease development was monitored and photographed periodically [29].

### 2.7. Appressorium Formation and Staining Analysis

Conidia of *C. orbiculare* were washed from CM plates with double-distilled water and adjusted to a concentration of 1 × 10^5^ conidia/mL to prepare the conidial suspension. A 20 μL aliquot of the conidial suspension was spotted onto a hydrophobic plastic membrane (Thermo Scientific, Waltham, MA, USA), followed by incubation at 22 °C in the dark. The morphology of appressoria was observed and photographed under a microscope at 4, 8, and 24 h post-inoculation (hpi), and the appressorium formation rate was calculated [30]. The experiment was performed in triplicate, with three technical replicates per trial.

Conidia of *Colletotrichum orbiculare* were diluted to a concentration of 1 × 10^5^ conidia/mL for appressorium induction. Glycogen and lipid droplet staining of conidia and appressoria was performed at 0, 4, 8, and 24 hpi. Glycogen was stained with a mixed KI/I_2_ solution (60 mg/mL KI and 10 mg/mL I_2_) for 1 min, followed by imaging and quantitative analysis under a light microscope [31]. Lipid droplets were stained with Nile Red (10 μg/mL) for 2 min and then observed under a fluorescence microscope (Zeiss Axio Image A2, Oberkochen, Germany). In the lipid droplet staining assay, tricyclazole (10 μg/μL) was added to the conidial suspension to inhibit melanin synthesis in appressoria, thereby facilitating the observation of fluorescence signals [32].

### 2.8. Phenotypic Analysis of Fungal Strains

Strains were grown on CM plates for 5 days at 28 °C. Mycelial plugs (5 mm in diameter) were taken from the colony margins and transferred onto fresh CM plates. Each strain was replicated five times. After incubation at 28 °C in darkness for 7 days, the growth rates were observed by measuring colony diameters, and photographs were taken. To assess sporulation capacity, 3 mL of sterile distilled water was added to each CM plate, and the surface of the colony was gently scraped with a sterile spreader to release conidia. The resulting suspension was filtered through three layers of sterile mesh to remove mycelial debris. Conidial concentration was determined using a hemocytometer, and the total sporulation yield of each strain was calculated and compared.

Mycelial plugs (5 mm in diameter) excised from 5-day-old colonies and inoculated onto CM agar plates amended with the following stress agents: osmotic stressors (0.1 M NaCl), oxidative stressors (5 mM CuSO_4_ and 25 μM Bengal rose), and cell wall disturbing agents (25 μg/mL Congo red and 50 μg/mL Calcofluor White). The plates were incubated at 28 °C in complete darkness for 7 days. Colony diameters were measured to assess stress adaptation capacity. The experiment was performed with three independent biological replicates, each containing five technical replicates.

Mycelial plugs (5 mm in diameter) were inoculated onto minimal medium (MM) [33] and glucose-free minimal medium (MM-C). In addition, lacking glucose but supplemented with each of the following carbon sources: 1% (*v*/*v*) Tween 80, 1% (*v*/*v*) olive oil, 50 mM sodium acetate (NaAC), or 10 mg/mL sucrose. Plates were incubated at 28 °C for 7 days; then, colony growth was measured to assess the ability of strains to utilize different lipid and alternative carbon sources.

### 2.9. Statistical Analysis

All quantitative data were obtained from at least three independent experiments, each with 3~5 technical replicates. Statistical differences between wild-type and mutant strains were analyzed by Student’s *t*-test at *p*-value < 0.01.

## 3. Results

### 3.1. Homology Analysis of CoPex8

**Figure 1:**
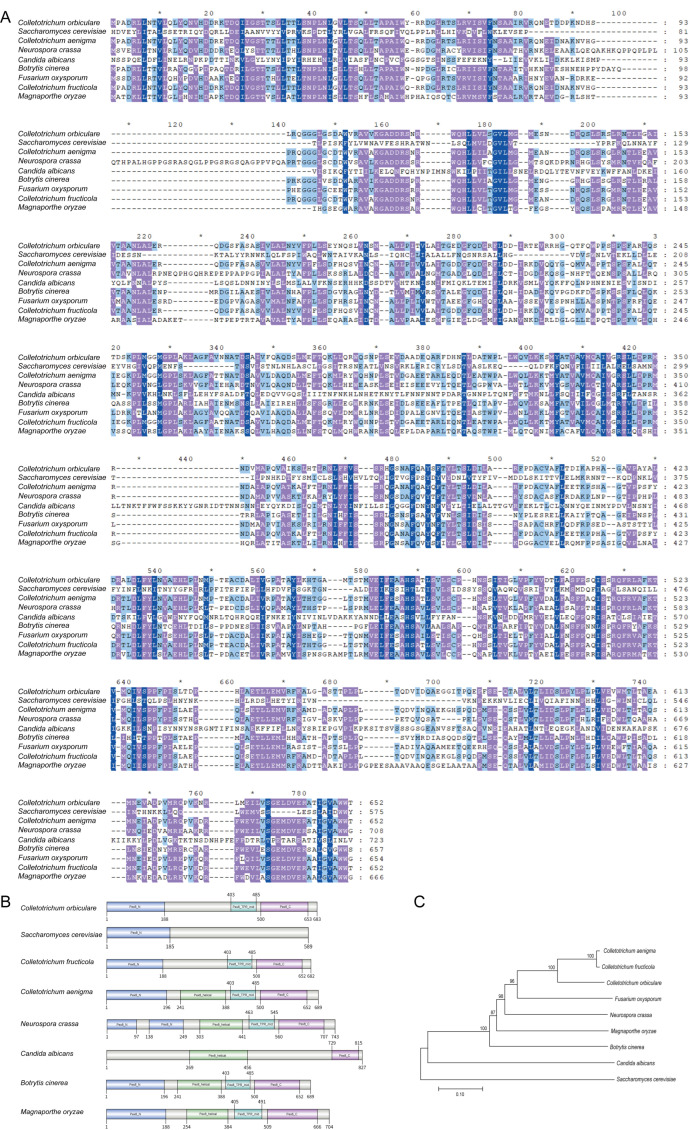
Homology analysis of CoPex8. (**A**) Multiple sequence alignment of CoPex8 with homologous proteins from related fungal species performed using Clustal X. Identical amino acids are highlighted with a dark blue background, conserved residues with a purple background, and similar residues with a light blue background. Positions with fully conserved residues are marked with asterisks. (**B**) Domain architectures of Pex8*.* (**C**) Phylogenetic tree of Pex8 homologs constructed with MEGA X based on the alignment in (**A**).

Using the amino acid sequence of BcPex8 from *B. cinerea* (BCIN_16g01260) as a query, homologous sequences of CoPex8 (Cob_v002892) from *C. orbiculare* were identified by Blastp searches in NCBI (https://www.ncbi.nlm.nih.gov/guide/proteins/, accessed on 3 October 2025). Multiple sequence alignment and Neighbor-Joining phylogenetic analysis confirmed that Pex8 is evolutionarily conserved across fungal species. CoPex8 protein shares 48.53% sequence homology with CaPex8 in *Colletotrichum aenigma*, 48.73% with MoPex8 in *Magnaporthe oryzae*, and 47.68% with BcPex8 in *B. cinerea* (Figure 1A,C). InterPro search (https://www.ebi.ac.uk/interpro/, accessed on 3 October 2025) revealed that CoPex8 had an N-terminal domain between amino acids (aa) 1 and 188, a Pex8 central TPR-like domain between aa 403 and 485, and a Pex8 c-terminal domain between aa 500 and 653 (Figure 1B). These domains are also conserved in other species.

### 3.2. CoPex8 Is Distributed on the Peroxisomal Membrane

**Figure 2:**
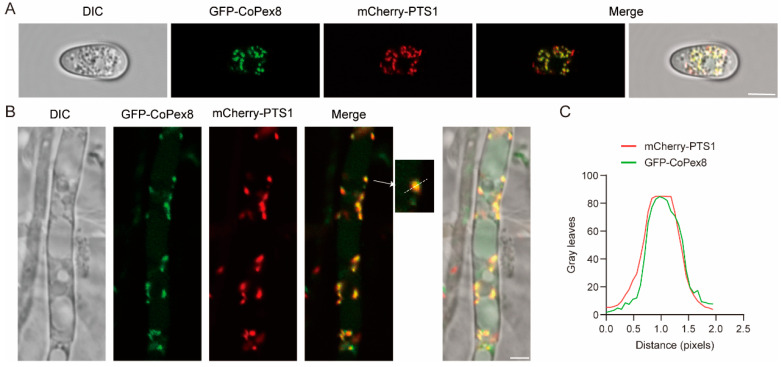
Peroxisomal localization of CoPex8 in *C. orbiculare*. (**A**) Colocalization of GFP-CoPex8 and mCherry-PTS1 in conidia. (**B**) Colocalization of GFP-CoPex8 and mCherry-PTS1 in hyphae, where the white arrows indicate the positions of the magnified fluorescent colocalization spots in the original image. Scale bar = 5 μm. (**C**) The fluorescence intensity profiles of GFP-CoPex8 and mCherry-PTS1 along the white dashed lines in (**B**) were analyzed using Image J 1.54g software.

Pex8 is a fungus-specific peroxisomal protein [10]. To determine the subcellular localization of CoPex8 in the peroxisome of *C. orbiculare*, we co-transformed a GFP-CoPex8 fusion protein-expressing vector and a mCherry-PTS1 fusion protein-expressing vector into the wild-type strain. The strains expressing GFP-CoPex8 and the peroxisomal matrix marker mCherry-PTS1 under the control of the MPG1 promoter were examined by confocal fluorescence microscopy. In both hyphae and conidia, GFP-CoPex8 exhibited a punctate distribution and colocalized substantially with mCherry-PTS1 (Figure 2A,B). In the enlarged images, GFP-Copex8 could be observed surrounding or closely adjacent to mCherry-PTS1 (Figure 2B,C). These results indicated that CoPex8 is localized on the peroxisomal membrane in *C. orbiculare*.

### 3.3. CoPEX8 Is Involved in Vegetative Growth and Conidiation in C. orbiculare

**Figure 3:**
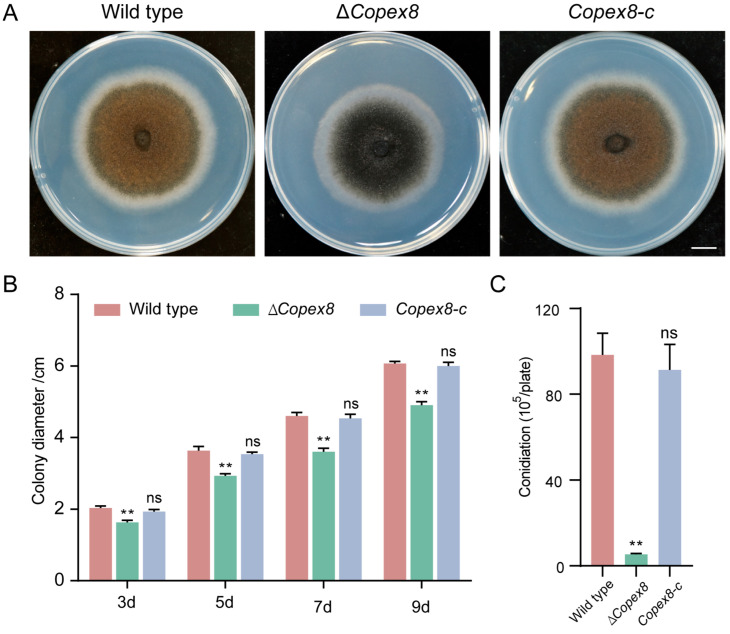
Growth and developmental defects in the Δ*Copex8* mutant. (**A**) Colony morphology of the wild-type, Δ*Copex8* mutant, and complemented strains cultured on CM for 9 days. Scale bar = 1 cm. (**B**) Mycelial diameters (cm) of the wild-type, Δ*Copex8* mutant, and complemented strains cultured on CM for 3–9 days. (**C**) Conidial yield of the wild-type, Δ*Copex8* mutant, and complemented strains. Data were analyzed by one-way ANOVA (ns: no significant difference; ** *p*-value < 0.01); scatter bars represent SDs.

The gene replacement strategy was employed to generate the Δ*Copex8* mutant (Figure S1). We analyzed the phenotype of the Δ*Copex8* mutant in fungal growth and conidiation on CM plates. Compared with the wild-type and complemented strain (*Copex8-c*), the mutant displayed sparse aerial hyphae and a 21.6% reduction in radial growth rate (Figure 3A,B). Moreover, the Δ*Copex8* mutant produced fewer conidia, with conidial yield reduced from approximately 9.83 × 10^7^ conidia/mL in the wild-type to 5.37 × 10^5^ conidia/mL in the mutant (Figure 3C). These results confirmed that CoPex8 is essential for normal hyphal growth and conidiation of *C. orbiculare*.

### 3.4. CoPex8 Is Required for Full Virulence of C. orbiculare

**Figure 4:**
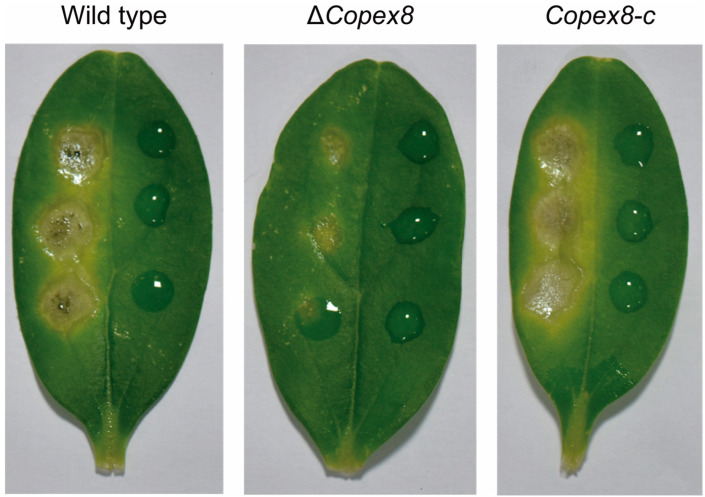
Attenuated virulence of the Δ*Copex8* mutant on cucumber leaves. Leaves were spray-inoculated with conidial suspensions (left half) or ddH_2_O (right half, mock control) and photographed at five days post-inoculation.

To investigate the function of *CoPEX8* in the virulence of *C. orbiculare*, conidia harvested from CM plates of the wild-type, Δ*Copex8* mutant, and complemented strains were suspended at 1 × 10^6^ conidia/mL and inoculated on the detached cucumber leaves. Five days post-inoculation, the wild-type and complemented strains produced expanding necrotic lesions, whereas the Δ*Copex8* mutant caused minimal symptoms, and its lesions expanded more slowly (Figure 4). Thus, we infer that CoPex8 is a key virulence factor in *C. orbiculare*.

### 3.5. CoPEX8 Is Essential for Appressorium Formation and Maturation

**Figure 5:**
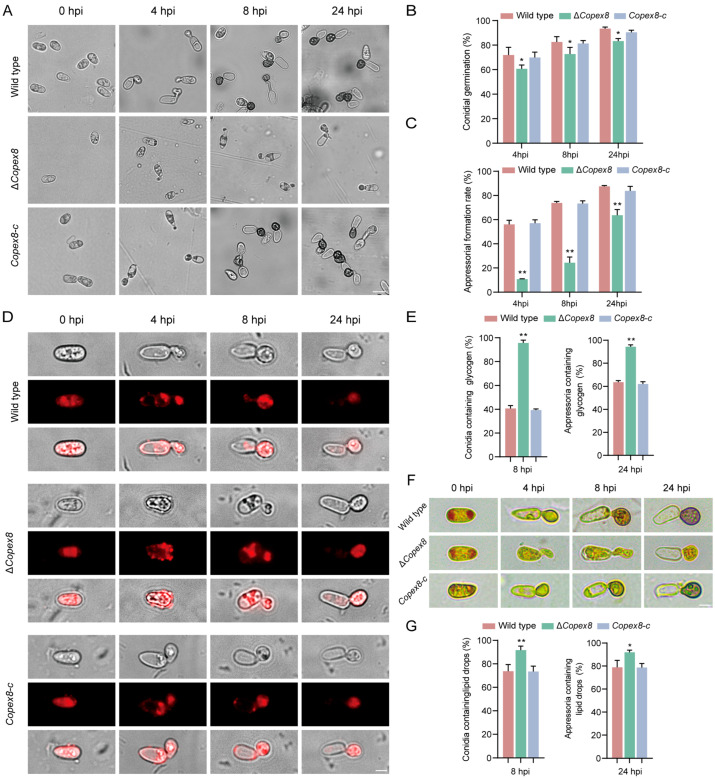
Defects in appressorium development and metabolite mobilization in the Δ*Copex8* mutant. (**A**) Conidial germination and appressoria morphology of the wild-type strain and Δ*Copex8* mutant. Scale bar = 10 μm. (**B**,**C**) Rates of conidial germination and appressorium formation. (**D**,**E**) Nile Red staining of lipid droplets. Scale bar = 5 μm. (**F**,**G**) Glycogen staining by I_2_/KI solution. Scale bar = 5 μm. Data were analyzed by chi-square test (* *p*-value < 0.05, ** *p*-value < 0.01); scatter bars represent SDs.

The dense melanin layer in the appressorium cell wall acts as a physical barrier to maintain the accumulation of high glycerol concentrations inside the cell, thereby generating substantial appressorium turgor pressure [12,34]. Conidial germination and appressorium formation were examined on hydrophobic surfaces. Conidial germination rates were similar between the wild-type and Δ*Copex8* strains. In contrast, appressorium formation was severely impaired: at 4 hpi, >58% of wild-type conidia formed appressoria versus ~10% for Δ*Copex8*; even at 24 hpi, the mutant reached only ~70% with delayed maturation (Figure 5A–C). In filamentous fungi, the generation of appressorium turgor pressure relies on the translocation and metabolism of glycogen and lipid droplets [14,15]. Mobilization of glycogen and lipid droplets, required for turgor generation, was examined during appressorium development. At 8 hpi, glycogen was largely degraded in the wild type (41% retention) but retained in 95% of Δ*Copex8* conidia. At 24 hpi, >94% of mutant appressoria still contained glycogen versus ~63% in the wild type (Figure 5D,E). Lipid droplet degradation showed a similar delay (Figure 5F,G). These results indicate that *CoPEX8* is required for timely metabolite translocation and degradation during appressorium maturation, thereby contributing to pathogenicity.

### 3.6. CoPEX8 Is Required for Peroxisome Biogenesis of C. orbiculare

**Figure 6:**
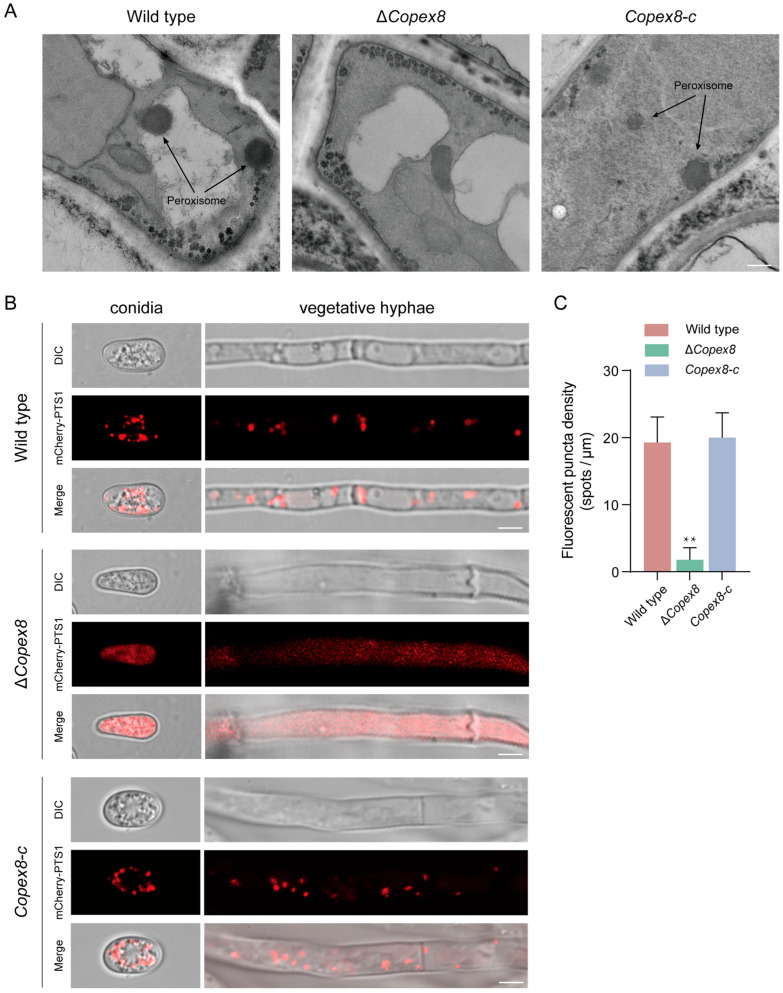
Absence of peroxisomes in the Δ*Copex8* mutant. (**A**) TEM micrographs of hyphal cells. Peroxisomes (black arrows) are present in the wild type but undetectable in the mutant. Scale bar = 0.25 μm. (**B**) Fluorescence of the peroxisomal marker mCherrymCherry-PTS1. Scale bar = 5 μm. (**C**) Calculation of the number of peroxisomes per unit area in fluorescent hyphae. Data were analyzed by chi-square test (** *p*-value < 0.01); scatter bars represent SDs.

To examine the impact of *CoPEX8* deletion on peroxisome structure, we compared the ultrastructure of the wild-type and Δ*Copex8* mutant strains by TEM analysis. In the wild type, well-defined peroxisomes (dark, rounded organelles) were abundant in hyphae cells, while mitochondrial cristae were clearly visible. In contrast, the Δ*Copex8* mutant exhibited no peroxisomes or peroxisome-like structures (Figure 6A). Thus, CoPex8 is essential for peroxisome biogenesis in *C. orbiculare.* Furthermore, we observed the import of peroxisomal matrix proteins marked with mCherry-PTS1 in both conidia and hyphae. Consistently, the mCherry-PTS1 showed punctate localization in the wild-type but diffuse cytosolic distribution in the Δ*Copex8* mutant (Figure 6B). Quantified the number of peroxisomes in fluorescent hyphae. The peroxisomal fluorescent puncta density per unit area was approximately 20 spots/μm in the wild-type and complemented strains, whereas it was only 2 spots/μm in the mutant strain (Figure 6C). Taken together, these data confirm that *CoPEX8* is indispensable for peroxisome formation of *C. orbiculare*.

### 3.7. CoPEX8 Is Required for Fatty Acid β-Oxidation and Free Oxidative Radicals Detoxification

**Figure 7:**
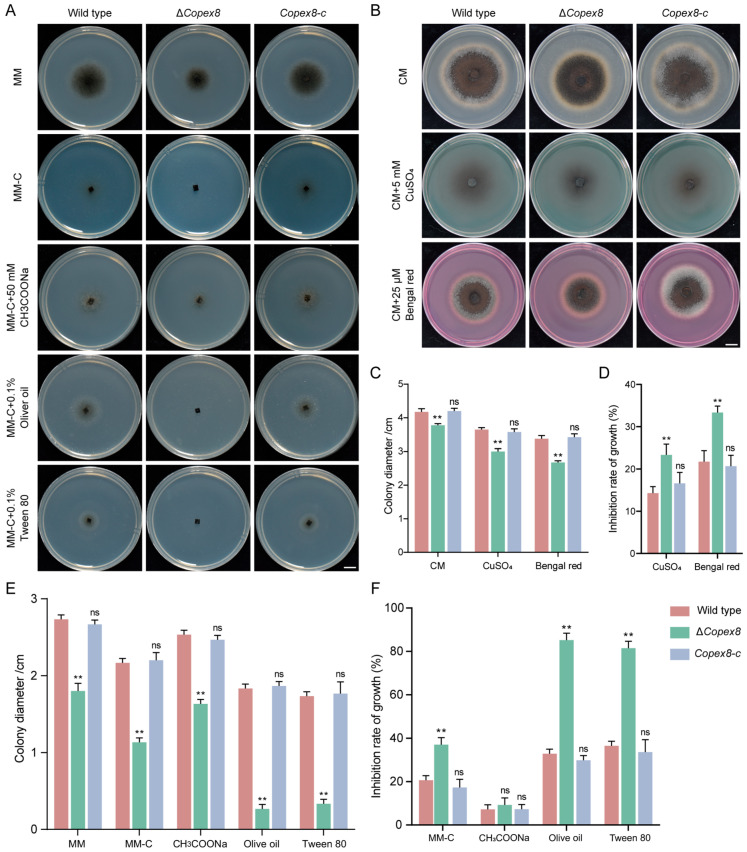
Impaired fatty acid utilization and ROS detoxification in the Δ*Copex8* mutant. (**A**,**E**,**F**) Growth of *C. orbiculare* strains on minimal media with different carbon sources. (**B**–**D**) Growth of *C. orbiculare* strains under oxidative stress. Scale bar = 1 cm. Data were analyzed by one-way ANOVA (ns: no significant difference; ** *p*-value < 0.01); scatter bars represent SDs.

Peroxisomes are the primary site of fatty acid β-oxidation and reactive oxygen species (ROS) detoxification in fungi [35]. To evaluate the effect of *CoPEX8* deletion on lipid metabolism, we measured the growth of the wild type, Δ*Copex8*, and Δ*Copex8-c* strains on minimal media (MM-C) supplemented with sodium acetate (CH_3_COONa) and fatty acids (Tween 80 or olive oil). The growth of the Δ*Copex8* mutant on minimal media containing Tween 80 or olive oil as the sole carbon source was significantly inhibited, with inhibition rates of 85.19% and 81.48%, respectively, whereas the inhibition rates of the wild-type strain were only 36.51% and 32.84%. However, the deletion of *PEX8* did not affect acetate utilization. These results indicate that the fatty acid β-oxidation of theΔ*Copex8* mutant was severely impaired (Figure 7A,E,F). To further investigate the ROS scavenging capacity of the mutant, the wild type, Δ*Copex8*, and Δ*Copex8-c* strains were cultured on CM supplemented with oxidative stress factors (CuSO_4_ and Bengal rose). The Δ*Copex8* mutant exhibited greater hypersensitivity to oxidative stress compared to the wild type. Specifically, the growth inhibition rates of the Δ*Copex8* mutant increased by 23.37% and 33.33% on CM supplemented with CuSO_4_ and Bengal red, respectively (Figure 7B–D). These results demonstrate that *CoPEX8* is required for peroxisome-dependent ROS detoxification in *C. orbiculare*.

### 3.8. CoPEX8 Influences Cell Wall Integrity, Osmotic Stress Response, and Melanin Biosynthesis

**Figure 8:**
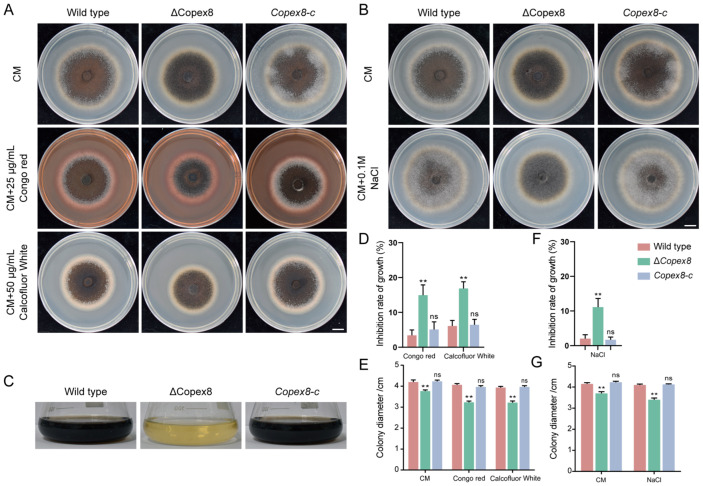
Compromised cell wall integrity, osmotic tolerance, and melanization in the Δ*Copex8* mutant. (**A**,**D**,**E**) Growth of *C. orbiculare* strains sensitive to cell wall stress. (**B**,**F**,**G**) Growth of *C. orbiculare* strains under NaCl stress. (**C**) Melanin production of *C. orbiculare* strains. Scale bar = 1 cm. Data were analyzed by one-way ANOVA (ns: no significant difference; ** *p*-value < 0.01); scatter bars represent SDs.

The pathogenicity of *C. orbiculare* is strongly correlated with melanin production, and cell wall integrity constitutes a critical prerequisite for melanin biosynthesis [36]. In this study, we assayed the sensitivity of the Δ*Copex8* mutant to cell wall-perturbing agents (Congo red, Calcofluor White), osmotic stress (NaCl), and oxidative stress (Methyl viologen). The results demonstrated that the mutant exhibited marked hypersensitivity to all tested stressors. Specifically, in the cell wall perturbation assays, the growth of Δ*Copex8* was significantly inhibited, with inhibition rates reaching 16.22% and 16.73%, respectively, whereas the inhibition rates of the wild-type strain were only 3.40% and 6.12% (Figure 8A,D,E). The hypersensitivity of the Δ*Copex8* mutant to osmotic stress increased by 10.09%, whereas the inhibition rate of the wild-type strain was only 2.04% (Figure 8B,F,G). In addition, melanin biosynthesis was drastically reduced in the mutant (Figure 8C). These findings indicate that deletion of the *CoPEX8* gene impairs cell wall integrity, reduces osmotic stress tolerance, and concomitantly inhibits melanin biosynthesis in the fungal strain.

## 4. Discussion

Peroxisome biogenesis has emerged as a critical determinant of virulence in several phytopathogenic fungi, including *M. oryzae*, *C. orbiculare*, and *Fusarium graminearum* [12,37,38]. Previous work from our laboratory and others has established that components of the peroxisomal import machinery, particularly the docking complex (Pex13, Pex14, Pex14/17) and the RING–finger complex (Pex2, Pex10, Pex12), are indispensable for development and pathogenicity in filamentous fungi [28,38,39]. In yeast, Pex8 functions as an intraperoxisomal peripheral membrane protein that bridges the docking and RING–finger complexes, thereby facilitating matrix protein import. Although the role of Pex8 is well characterized in yeasts, its function in filamentous fungi has remained largely unexplored [21,24]. Here, we provide the comprehensive characterization of Pex8 in a plant pathogenic fungus and show that CoPex8 is required for peroxisome biogenesis and multiple virulence-associated processes in *C. orbiculare*. We demonstrate that *CoPEX8* is essential for vegetative growth, conidiation, appressorium development, peroxisome biogenesis, lipid metabolism, redox homeostasis, cell wall integrity, and pathogenicity in *C. orbiculare*.

In recent years, peroxisomal metabolism has been demonstrated to be a key determinant of pathogenicity in several phytopathogenic fungi. *PEX5*, *PEX7*, *PEX13*, and *PEX14* are required for the development and pathogenicity of *M. oryzae* [38,39,40]. *PEX13* has been identified as an indispensable factor for the infection of *Colletotrichum orbiculare* [12]. In yeast, Pex8 has been verified as a peroxisomal membrane-associated peroxin that mediates protein translocation across the peroxisomal membrane, which is essential for the correct subcellular localization of proteins carrying PTS1 and PTS2 [21]. Ultrastructural observations showed that, in contrast to the wild-type strain, the hyphae of the Δ*Copex8* mutant contained very few regular spherical peroxisomes. This finding is consistent with the phenotype observed in *M. oryzae* after deletion of other peroxisome-related genes, indicating that the knockout of *CoPEX8* inhibits peroxisome biogenesis [39]. Meanwhile, the absence of *CoPEX8* led to the cytoplasmic distribution of mCherry-PTS1, which failed to be properly imported into peroxisomes [28]. Pex8 contains a conserved C-terminal motif that interacts with the N-terminal domain of the PTS1 receptor Pex5 within the peroxisomal lumen [22]. Under reducing conditions, Pex8 promotes conformational changes in Pex5, reduces its affinity for PTS1 cargo, and facilitates cargo release [41]. Pex8 also contributes to Pex5 and recycling, ensuring sustained matrix protein import [20,22]. Thus, Pex8 acts as a critical regulator that links receptor-cargo dissociation with receptor export, thereby maintaining the directionality and efficiency of the PTS1 import cycle.

During the process of pathogen invasion and colonization, the host produces a substantial amount of ROS to defend against the invasion. For the pathogen to successfully parasitize the host, it must overcome the impact of ROS, and the degradation of ROS also relies on peroxisomes [42]. The hypersensitivity of the Δ*Copex8* mutant to exogenous oxidants indicates that peroxisome-dependent catalase and other antioxidative enzymes are non-functional, leading to intracellular ROS accumulation and oxidative damage. This redox imbalance likely exacerbates the observed defects in growth, conidiation, and infection structure differentiation [42]. Because peroxisomal is the primary route for fatty acid catabolism in fungi [19], the near-complete growth arrest of Δ*Copex8* on media containing Tween-80 or olive oil as sole carbon sources directly reflects the absence of functional peroxisomes. Cell wall integrity and melanin biosynthesis were similarly compromised in the absence of *CoPEX8*. Peroxisomal β-oxidation supplies acetyl-CoA for the glyoxylate cycle and subsequent carbohydrate synthesis, which provides precursors for cell wall polysaccharides and melanin [6,42]. Impaired import of glyoxylate cycle enzymes in pex mutants is known to disrupt these pathways, consistent with the hypersensitivity to cell-wall-perturbing agents and reduced melanization observed here [2].

The PTS1 and PTS2 transport pathways—mediated by Pex5 and Pex7, respectively—are functionally complementary, yet each has its own emphasis. In the rice blast fungus *M. oryzae*, the role of the PTS1 pathway mediated by MoPex5 is more prominent than that of the PTS2 pathway mediated by MoPex7 [6,42]. As a key peroxin regulating peroxisome biogenesis in *C. orbiculare*, CoPex8 has been confirmed to affect the targeting process of peroxisomal targeting signal 1 (PTS1) to peroxisomes. However, it remains to be further confirmed whether *CoPEX8* is involved in the regulation of the peroxisomal targeting signal 2 (PTS2) pathway, as well as how it cooperates with the docking complex and the RING-finger complex in the import process of peroxisomal matrix proteins. Subsequent experiments will focus on elucidating the regulatory role of *CoPEX8* in the PTS2 pathway and its mechanism of synergistic interaction with the two complexes, so as to fill the existing research gaps and improve the CoPEX8-mediated regulatory network of peroxisomal functions.

## 5. Conclusions

**Figure 9:**
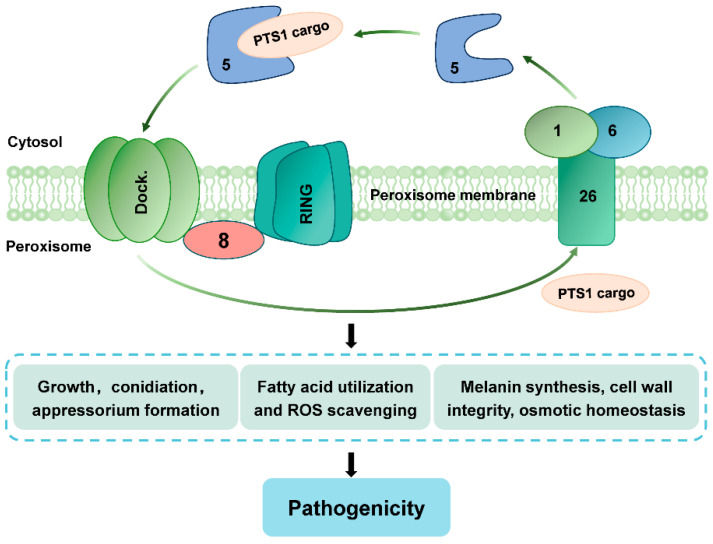
Schematic diagram for the functional role of CoPex8 in *C. orbiculare*. CoPex8 is localized on the peroxisomal membrane and is involved in the import of peroxisomal matrix proteins. The numbers in the diagram indicate: 8: CoPex8, 5: CoPex5, 1: CoPex1, 6: CoPex6, 26: CoPex26. CoPex8 regulates the pathogenicity of *C. orbiculare* by modulating fungal growth, appressorium development, fatty acid utilization, ROS scavenging, melanin synthesis, cell wall integrity, and osmotic homeostasis.

*CoPEX8* is an essential peroxin in *C. orbiculare* that orchestrates peroxisome biogenesis and thereby sustains lipid catabolism, ROS homeostasis, carbon metabolite mobilization, cell wall integrity, and melanin production, all processes indispensable for appressorium-mediated host invasion. These findings highlight the pivotal role of fungal-specific peroxins in plant pathogenesis and reinforce peroxisomes as promising targets for novel antifungal strategies (Figure 9).

## Data Availability

The original contributions presented in this study are included in the article/Supplementary Material. Further inquiries can be directed to the corresponding author.

## Supplementary Materials

The following supporting information can be downloaded at: https://www.mdpi.com/article/10.3390/jof12040248/s1, Figure S1: Deletion of *CoPEX8* gene in *C. orbiculare*; Table S1: Primers used in this study.
