# Supplementary material for: Pex8, a Fungal Specific Peroxin, Regulates Peroxisome Biogenesis and Pathogenicity in the Cucumber Anthracnose Fungus Colletotrichum orbiculare
Source: J Fungi (Basel). 2026 Mar 30;12(4):248. doi: 10.3390/jof12040248 (PMC13117297; doi:10.3390/jof12040248)
Supplement: Supplementary file 1 [file jof-12-00248-s001.zip › jof-4165731-supplementary.pdf]

Journal of Fungi

MDPI

Supplementary Materials

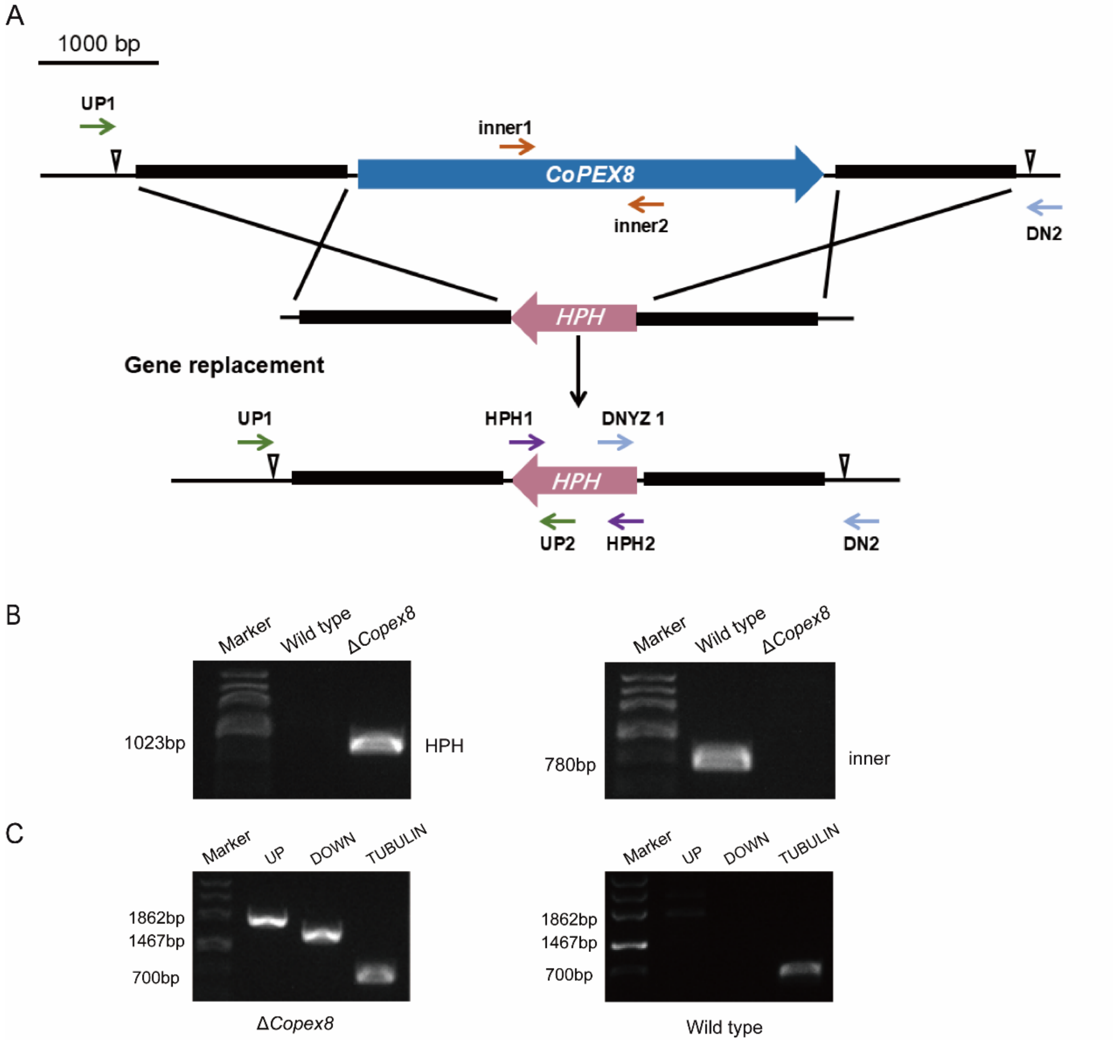

Figure S1. Deletion of *CoPEX8* gene in *C. orbiculare*. (A) Schematic diagram of the gene knockout strategy. *CoPEX8* coding region was replaced by the hygromycin-resistance gene (*HPH*) cassette. The positions of the primers are indicated by arrows, with primers of the same color used as a pair. (B) Validation of *CoPEX8* knockout. The primer set (CoPEX8 inner-F/CoPEX8 inner-R) was used to verify the target gene, and the primer set (HPH-F/HPH-R) was used to amplify the fragment of *HPH* in mutant. (C) PCR verification of the recombinant DNA fragment (amplified by primers CoPEX8 upyz-F/CoPEX8 upyz-R or CoPEX8 downyz-F/CoPEX8 downyz-R), with *β-TUBULIN* DNA used as a positive control.

Table S1. Primers used in this study.

Primer	Sequence (5′-3′)
CoPEX8 up-F	AGGCTAACTGACACTCTAGATTCTCATAAGCCACCCTGAATT
CoPEX8 up-R	ATGCCTGCAGGTCGACTCTAGACGCATCACCCCGTCGCAACC
CoPEX8 down-F	CCGAGGGCAAAGGAATAGGAGCTCACGTGGTGGCAACAGCATA

CoPEX*8* down-R	GCTATGACCATGATTACGAATTCCTCTTATTGTATCTCGCCCGC
CoPEX8-F	GACGAGCTGTACAAGTCTAGAATGCCGGCCGACCGACTTCTCAAC
CoPEX8-R	GCTATGACCATGATTACGAATTCCTCTTATTGTATCTCGCCCGC
CoPEX8 upyz-F	GCTCCTTCTACAGCGGCACACA
CoPEX8 upyz-R	GGTCTTGCAACGTGACACCCTG
CoPEX8 downyz-F	CCGGGCGTATATGCTCCGCAT
CoPEX8 downyz-R	GCGGGGCCAACGTCAATCGTA
CoPEX8 inner-F	ATGCCGGCCGACCGACTTCTC
CoPEX8 inner-R	TCAAAGCCTGCTCATCGTCCC
HPH-F	TAGTGGAGGTCAACAATGAATG
HPH-R	CATCTACTCTATTCCTTTGCCC
TUB-F	AACATGCGTGAGATTGTAAGT
TUB-R	ACCCTCAGTGTAGTGACCCTTGGC
MPG11	TCACGACTGGGAGTAGAAAGA
MPG12	CCAGATTCCAGGGTTGCTAAA
GFP-CHK1	GCCACCTACGGCAAGCTGACCCTG
GFP-CHK2	GGGTGCTCAGGTAGTGGTTGTCGG
RED1	CCGGGGTGAGCAAGGGCGAGG
RED2	TCTAGAGCCGCCGGTGGAGTG
NEO1	CGGGTAGCCAACGCTATGTCC
NEO2	GGACGACCCGGTCATACCTTC
H3-1	AGTCATGTTGATTGAGGTGT
H3-2	GGCCATTGTGATTGATTTGTG
pNMCherryA1	TCGGCGCGTTCGTACTGTTCC
PNMCherryA2	ATGGTGAGCAAGGGCGAGGAG

https://doi.org/10.3390/xxxxx
